# Can Overuse of Kambô Cause Psychosis?

**DOI:** 10.7759/cureus.2770

**Published:** 2018-06-09

**Authors:** Renitha Roy, Aparna Baranwal, Eduardo D Espiridion

**Affiliations:** 1 Frederick Memorial Hospital, West Virginia School of Osteopathic Medicine, Lewisburg, USA; 2 Medical Student, West Virginia School of Osteopathic Medicine, Lewisburg, USA; 3 Psychiatry, Frederick Memorial Hospital, Frederick , USA

**Keywords:** phyllomedusa bicolor, kambô, toxin-induced psychosis, giant leaf frog toxin, shaman ritual, neurochemical effects of toxin

## Abstract

Kambô is an emerging ritual, which involves the application of the toxin produced by Phyllomedusa bicolor to a freshly burnt area to heal chronic diseases of the mind and body. Due to the widespread use of kambô, more cases of symptomatic health conditions are being discovered. In this case study, we report a patient with psychosis potentially due to the kambô ritual.

## Introduction

Kambô is an emerging ritual, which involves the application of a toxin produced by a giant leaf frog, Phyllomedusa bicolor, to a freshly burnt skin area. This is being used to heal the chronic diseases of the mind and body. Due to the widespread use of kambô, more cases of symptomatic health conditions are being observed. In this case report, we report a patient who presented with new-onset psychotic symptoms, potentially due to the kambô ritual.

## Case presentation

The patient is a 33-year-old Caucasian female, who was brought to the local emergency room by the police. The police were repeatedly called by the patient about rapes and shootings in her community. On the day she was brought to the hospital, the patient called the police under a fake name and complained that her husband was raping another individual. She was making nonsensical comments, including being ritualistically haunted by her father and sister. The patient was found to be unmarried and lived alone but was adamant about being married to a celebrity. She had no significant psychiatric history prior to this incident. After acknowledging that she was a certified shaman and practices healing through the utilization of the kambô ritual, she claimed that she uses the kambô toxin to alleviate her chronic pain. Her frequency of performing the ritual changed from once per month to up to nine times per month. She presented with characteristics of paranoia, anxiety, bizarre delusions, labile mood, and panic attacks. On physical examination, scars were noted on the patient’s legs from the burns and administration of the toxin. She subsequently had an unremarkable extensive medical workup. As part of her treatment plan, the patient was started on risperidone and she gradually improved after nine days in the hospital psychiatry unit.

## Discussion

Phyllomedusa bicolor is a giant leaf frog that secretes toxins that potentially contain peptides, such as phyllokinin, phyllocaerulein, phyllomedusin, sauvagine, deltorphins, dermorphins, and adrenoregulin.

This particular venom is used in a ritual of applying the toxins to a freshly inflicted burn on the body. The rituals are meant to purify the human body. However, there have been case reports of toxic hepatitis, syndrome of inappropriate antidiuretic hormone secretion (SIADH), and even death [1-3]. The toxin is found to have rapid effects of tachycardia, vomiting, and incontinence, which have led to euphoria and sedation [4].

Many components of the toxin have been discovered to have neurochemical effects. Deltorphin is a full selective agonist for the delta opioid receptors in the central nervous system. It has shown an analgesic effect in mice through the morphine (mu) and ketocyclazocine (kappa) opioid receptors [5]. Moreover, dermorphin has been shown to have a high potency and selectivity to the mu opioid receptors but has weak anti-nociceptive properties [6]. Another constituent, sauvagine, is associated with behavioral effects in mice. Sauvagine has effects on the adrenal cortex and corticotrophin-releasing factor (CRF). Data has shown that patients with post-traumatic stress disorder (PTSD) with secondary psychosis had increased levels of CRF, but in the control group with PTSD and no psychosis, CRF levels were within normal limits [7-8]. The patient had multiple doses of the kambô toxin leading to her hospital admission, which potentially had increased her CRF and may have caused her psychotic symptoms. With the various neuropeptides in the kambô toxin, which have known and unknown effects and interactions, any combinations of these compounds could have led to her psychotic status.

## Conclusions

This is a patient who presented with significant disorganized behaviors and eccentric delusions without any known psychiatric issues. The only significant information pertaining to her was an increased use of the kambô toxin and her symptoms responded to antipsychotics. Based on the various components of the toxin, there can be a correlation between her psychosis and the overuse of kambô. Even with a recent increase in reported health impacts, there is still uncertainty on the complex toxic effects of the kambô toxin. Since these rituals have been increasing worldwide, such clinical presentations may increase as well.
